# Dependence of Present and Future European Temperature Extremes on the Location of Atmospheric Blocking

**DOI:** 10.1029/2018GL077837

**Published:** 2018-06-28

**Authors:** Lukas Brunner, Nathalie Schaller, James Anstey, Jana Sillmann, Andrea K. Steiner

**Affiliations:** ^1^ Wegener Center for Climate and Global Change University of Graz Graz Austria; ^2^ FWF‐DK Climate Change University of Graz Graz Austria; ^3^ Now at Institute for Atmospheric and Climate Science, ETH Zurich Zurich Switzerland; ^4^ Center for International Climate Research (CICERO) Oslo Norway; ^5^ Canadian Centre for Climate Modelling and Analysis, Environment and Climate Change Canada University of Victoria Victoria British Columbia Canada; ^6^ Institute for Geophysics, Astrophysics, and Meteorology, Institute of Physics University of Graz Graz Austria

**Keywords:** atmospheric blocking, heat waves, cold spells, Europe, climate model, large ensemble

## Abstract

The impact of atmospheric blocking on European heat waves (HWs) and cold spells (CSs) is investigated for present and future conditions . A 50‐member ensemble of the second generation Canadian Earth System Model is used to quantify the role of internal variability in the response to blocking. We find that the present blocking‐extreme temperature link is well represented compared to ERA‐Interim, despite a significant underestimation of blocking frequency in most ensemble members. Our results show a strong correlation of blocking with northern European HWs in summer, spring, and fall. However, we also find a strong anticorrelation between blocking and HW occurrence in southern Europe in all seasons. Blocking increases the CS frequency particularly in southern Europe in fall, winter, and spring but reduces it in summer. For the future we find that blocking will continue to play an important role in the development of both CSs and HWs in all seasons.

## Key Points

- Blocking strongly impacts both heat waves and cold spells in Europe depending on region and season
- Blocking‐extreme temperature link is found realistic in the model despite its underestimation of blocking
- Blocking will continue to play a crucial role for extreme temperatures also in the future

## 1 Introduction

There is ever increasing evidence that anthropogenic climate change affects society through an increase in extreme weather events and will continue to do so in the future (IPCC, 2013; Fischer & Knutti, 2015). Understanding the physical mechanisms behind high‐impact extremes such as heat waves (HWs) or cold spells (CSs) is therefore essential for policy makers in order to be able to take informed decisions (Zhang, 2013). One of the main drivers of European weather and climate variability is the frequent occurrence of atmospheric blocking (Woollings, 2010). Blocking describes strong and stationary high‐pressure systems, which interrupt the climatological flow at midlatitudes for up to several weeks (Rex, 1950). They are often linked to the development of surface HWs (e.g., Pfahl & Wernli, 2012) due to their persistence, which allows the accumulation of heat in a certain area (Bieli et al., 2015; Perkins, 2015). The Russian HW in summer 2010 is one recent example of a block leading to a devastating HW, which affected large parts of Europe and Russia. The exceptionally hot temperatures, which lasted for several weeks, led to over 50,000 additional deaths and billions of US Dollars in economic losses in Russia alone (e.g., Barriopedro et al., 2011). The role of the extraordinary atmospheric conditions caused by the blocking in the development and maintenance of the Russian HW was discussed by many studies in the aftermath (e.g., Galarneau et al., 2012; Lupo et al., 2012; Miralles et al., 2014; Schneidereit et al., 2012). Conversely, blocking has also been connected to winter CSs (e.g., Buehler et al., 2011; Sillmann et al., 2011), mainly through the advection of cold air (e.g., Bieli et al., 2015; Sousa et al., 2018). Recently, also the blocking impacts during the transition seasons such as spring have come into focus (e.g., Brunner et al., 2017; Cassou & Cattiaux, 2016). Extreme temperatures in spring can have strong impacts on vegetation, as plants are particularly vulnerable during the early green‐up phase (e.g., Hufkens et al., 2012). In spring 2016, for example, a blocking event over the British Isles led to a CS in central and eastern Europe, causing severe damages to agricultural yields in several countries with harvest failures of up to 80% (AGRI4CAST, 2016).

Reliable knowledge on the impacts of blocking in a changing climate is therefore of utmost importance for our understanding of the development of both HWs and CSs (IPCC, 2013). However, some of the processes involved in the blocking development and maintenance are still not well understood and trends in the future evolution of blocking remain uncertain (IPCC, 2013; Woollings, 2010). In the last decades many studies have noted the limited model performance in realistically representing blocking (e.g., Anstey et al., 2013; D'Andrea et al., 1998; Davini & D'Andrea, 2016; Schiemann et al., 2017). Davini and D'Andrea (2016) find that several models have significantly improved their representation of blocking in recent years. However, the blocking frequency in the Euro‐Atlantic sector is still underestimated by as much as 50% in the most recent Coupled Model Intercomparison Project Phase 5 (CMIP5) models (Davini & D'Andrea, 2016). For the future a decrease in blocking frequency and magnitude in the Euro‐Atlantic sector has been predicted, depending on the season (Kennedy et al., 2016; Matsueda & Endo, 2017). These considerations raise the question of the representation of the link between blocking and extreme temperature occurrence in models. Masato et al. (2014) investigate the impact of winter blocking on European temperature extremes during present and future conditions. They find that the models included in their study represent the relationship between blocking and temperature extremes well, given the negative bias in the blocking frequency. However, many blocking impact studies in the past have been based on only a few simulations and have focused on either the winter or summer season (e.g., Kennedy et al., 2016; Masato et al., 2014).

In this study we investigate all seasons and particularly also consider spring and fall. We use a large‐ensemble approach that provides enough cases to draw from in order to allow an estimation of uncertainty due to internal variability, given that blocking is a rare phenomenon. Our study looks into the relationship between both extreme cold and extreme warm temperatures and blocking in a 50‐member ensemble of the the second generation Canadian Earth System Model (CanESM2) (e.g., Fyfe et al., 2017). Our work has two main aims: (i) We evaluate the performance of the CanESM2 ensemble in representing present blocking and its link to temperature extremes compared to the ERA‐Interim reanalysis (Dee et al., 2011). (ii) We investigate how present and future temperature extremes in northern and southern Europe depend on the region of the blocking and on the season.

This work is structured as follows. Section 2 describes data sets and methods used in this study. Blocking frequencies in present and future conditions are addressed in section 3, and blocking impacts on European temperature extremes are presented in section 4. A concluding discussion is given in section 5.

## 2 Data and Methods

### 2.1 Data Sets

As reference data set in this study we use the ERA‐Interim reanalysis (Dee et al., 2011) at 0.75° × 0.75° spatial resolution and for the period 1981–2010. To assess the representation of the link between blocking and extreme temperatures as well as potential changes in the future, we consider a 50‐member initial‐condition ensemble from the CanESM2 model (Canadian Sea Ice and Snow Evolution project). The model ensemble data are available from 1950 until 2100, and we use the period 1981–2010 as present and 2070–2099 as future. The present and future periods of the simulations are driven by the CMIP5 historical and Representative Concentration Pathway 8.5 forcing scenarios, respectively. The 50 ensemble members differ only in their initial conditions; the forcing and model configuration are identical for each ensemble member. CanESM2 is a coupled atmosphere‐ocean climate model that includes sea ice, land, and carbon cycle components. The atmospheric model is horizontally spectral with T63 resolution, corresponding roughly to a grid spacing of 2.5° × 2.5°. For further information on the CanESM2 model and the construction of the 50‐member large ensemble, see Arora et al. (2011) and Fyfe et al. (2017).

### 2.2 Blocking Index

We use a standard two‐dimensional blocking detection algorithm based on the reversal of 500‐hPa geopotential height gradients (Anstey et al., 2013; Rex, 1950; Scherrer et al., 2006; Tibaldi & Molteni, 1990). At each grid point geopotential height gradients to the north (ΔZ_N_) and to the south (ΔZ_S_) are calculated as

(1) $$\begin{array}{ccc} \Delta Z_{N} & =\frac{Z(\lambda ,\phi +\Delta \phi )-Z(\lambda ,\phi )}{\Delta \phi } & \\ \Delta Z_{S} & =\frac{Z(\lambda ,\phi )-Z(\lambda ,\phi -\Delta \phi )}{\Delta \phi }, \end{array}$$

where Z is the geopotential height at 500 hPa, Δϕ = 15°, and ϕ ranges from 50°N to 75°N. Instantaneous blocking (IB) is defined at a certain grid point if the gradients simultaneously fulfill Z_N_<−10m/(° latitude) and Z_S_>0m/(° latitude). Spatiotemporal filtering following Woollings et al. (2008) is then applied to the IB field to select only large‐scale and slow‐moving cases. The maximum of the IB index over ±4° latitude is taken to allow for some meridional movement. Then extended IB cases are selected if they extend over at least 15° longitude to filter out too small systems. Finally, blocking is defined if extended IB is found within ±10° longitude for at least five consecutive days in order to detect only persistent and stationary systems.

Blocked days are further defined for three 30° longitude regions, referred to as the Greenland (60°W to 30°W), North Atlantic (30°W to 0°), and Scandinavian (0° to 30°E) regions. A day is considered as blocked in a given region if a block extends over more than half of the region (i.e., over more than 15° of longitude inside the region).

### 2.3 Heat Waves and Cold Spells

Two types of temperature extremes are considered on a daily basis for European land grid points. A HW is defined as a period of at least three consecutive days with daily maximum temperature above the maximum temperature threshold at a certain grid point. This threshold is defined for each day as the 90th percentile of the climatological distribution of daily maximum temperature at the grid point in one of the two periods during a ±15‐day window centered on the calendar day. Equivalently, a CS is defined as a period of at least three consecutive days with daily minimum temperature below the minimum temperature threshold. Analogously to the HW case, the minimum temperature threshold is defined as the 10th percentile of climatological minimum daily temperature (e.g., Russo et al., 2015).

Climatological HW/CS frequencies as well as the HW/CS frequencies during blocked days are calculated on a grid point basis for the two 30‐year periods representing present and future conditions. For both periods the relative anomaly is then computed for each grid point following:

(2) $$x_{\text{anom}}=\frac{x_{\text{blocking}}}{x_{\text{clim}}},$$

where x_blocking_ is the HW/CS frequency during blocked days and x_clim_ is the climatological frequency during all days (including blocked days).

We also calculate the mean HW/CS anomaly on the land surface in two regions: northern Europe (15°W to 30° E and 50°N to 75°N), and southern Europe (15° W to 30°E and 35°N to 50°N). Since we show the anomalies as multiples of the climatological frequency, single outlier grid cells (where x_blocking_ is very small) can have potentially large impacts on the area mean in this view. We therefore restrict the anomalies to decreases by one fifth and increases by a factor 5 for the calculation of the area mean.

### 2.4 Significance Testing

To establish the statistical significance of the link between blocking and HWs/CSs, we use a Monte Carlo test. For a certain number of blocked days N in a given setting (i.e., region, period, season, and ensemble member) the same number of N random days is drawn. To conserve autocorrelation, consecutive blocked days are clustered and lead to clusters of the same size in the random samples. The random draw is repeated 100 times, and from this distribution we use the 5th and 95th percentiles as thresholds for statistical significance. A certain value can therefore be either statistically significantly higher or lower, or not significantly different. In the figures showing the ensemble mean, the percentage of ensemble members showing statistical significance is indicated. Only values with no contradiction (i.e., one member showing a significantly higher and another member a significantly lower value) in the significance measure are indicated.

## 3 Blocking Frequencies in Present and Future

**Figure 1:**
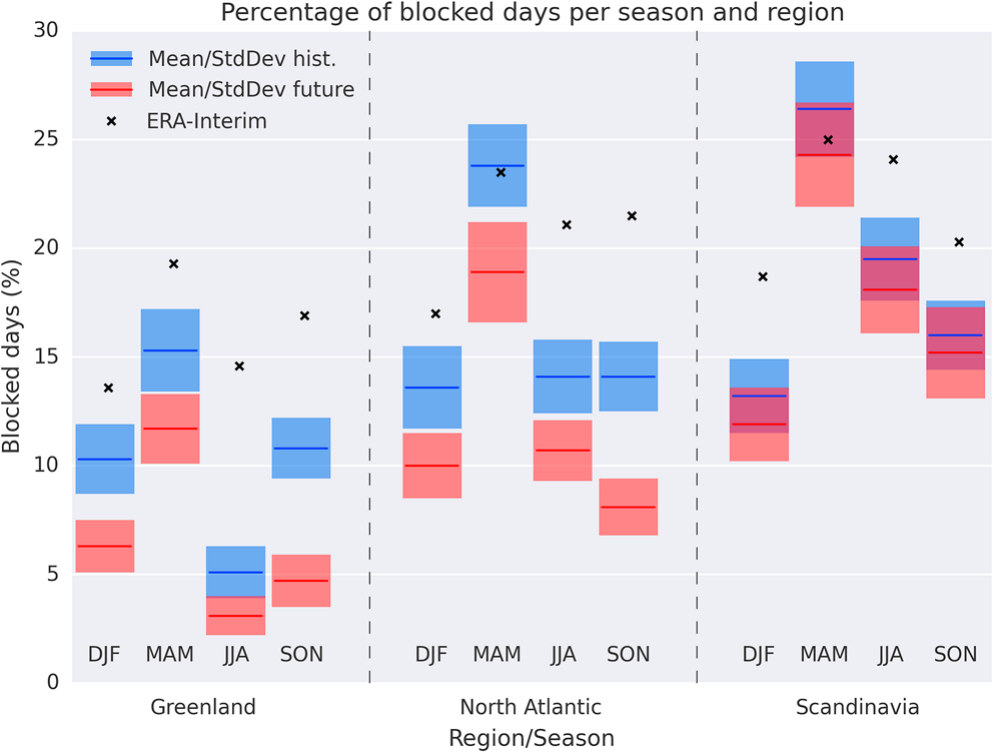
Percentage of blocked days per region and season for ERA‐Interim (× signs) and the CanESM2 ensemble mean (colored lines) and ensemble standard deviation (colored boxes) during present (blue) and future (red) conditions (see Table S1 in the supporting information for underlying statistics). CanESM2 = second generation Canadian Earth System Model; DJF = December–February; MAM = March–May; JJA = June–August; SON = September–November.

We first investigate the present‐day blocking representation in the CanESM2 ensemble members and compare it to ERA‐Interim. Figure 1 shows the relative number of blocked days in all regions and seasons for ERA‐Interim and the CanESM2 ensemble mean and standard deviation. For most seasons and regions the number of blocked days is underestimated by the model mean by up to a factor 2 or more compared to ERA‐Interim. Only for the North Atlantic and Scandinavia regions in spring the ERA‐Interim blocking frequency lies within one standard deviation of the CanESM2 ensemble mean. For the future, we find a strong decrease in the number of blocked days across all seasons and regions in agreement with recent literature (e.g., Kennedy et al., 2016; Matsueda & Endo, 2017). However, we notably do not find any significant change in the Scandinavian region, which is in line with Masato et al. (2014), who note that the decrease of blocking occurrence in the Euro‐Atlantic region strongly depends on the selection of the region and may rather be a shift to the east. Indeed, additionally investigating the region from 30°E to 60°E reveals a slight future increase in the blocking frequency in summer and fall.

## 4 Blocking Impacts

### 4.1 Blocking Impacts on Present Winter Cold Spells

**Figure 2:**
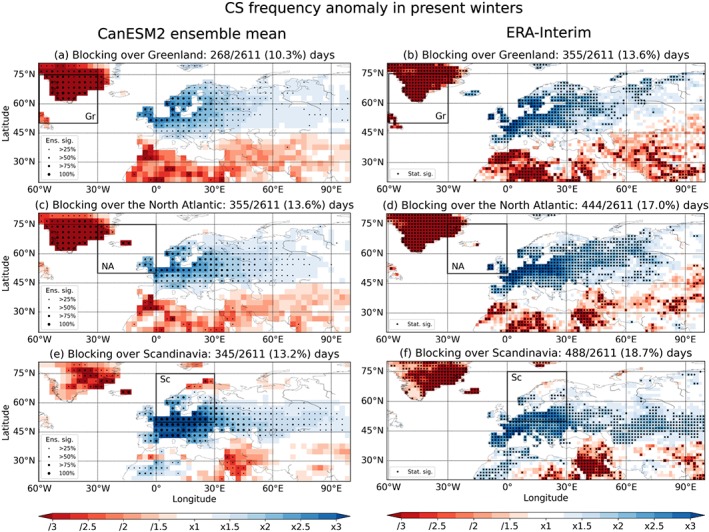
Cold spell frequency anomaly during blocking in different regions (black box) for winters (December–February) in the period 1981 to 2010 for the CanESM2 ensemble mean (a, c, e) and ERA‐Interim (b, d, f). Statistical significance at the 10% (two‐sided) level is indicated by dots. The larger the dot size, the larger is the number of ensemble members that show statistical significance for CanESM2. Gr = Greenland; NA= North Atlantic; Sc = Scandinavia; CanESM2 = second generation Canadian Earth System Model.

The link between blocking in the three different regions and the CS frequency in present‐day European winters (December–February) is shown in Figure 2. Despite the considerable discrepancy in the blocking representation discussed above, the extreme temperature response to blocking in all three regions is remarkably similar in the CanESM2 ensemble mean compared to ERA‐Interim, in line with, for example, findings of Masato et al. (2014). Blocking over Greenland leads to a strong and statistically significant increase in the CS frequency across most of Europe, which is consistent between CanESM2 and ERA‐Interim (Figures 2a and 2b). For blocking in the North Atlantic and Scandinavian regions the anomalies shift toward central Europe, while the influence in Scandinavia decreases (Figures 2c–2f). Particularly during blocking in the Scandinavian region, it is noteworthy that most of the 50 ensemble members show a more than threefold, statistically significant increase in the CS frequency in central Europe, in agreement with ERA‐Interim (Figures 2e and 2f). This strong cooling effect of blocking in winter has been found to be dominantly driven by the advection of cold air (Bieli et al., 2015; Pfahl, 2014; Sousa et al., 2018). Bieli et al. (2015) calculated backward trajectories to evaluate the source region of air masses connected to cold extremes. For example, for central Europe they found that the regions of origin are mainly located to the north and east, which is consistent with advection due to blocking anticyclones. Conversely, Sousa et al. (2018) showed that for blocking located over the North Atlantic and Scandinavian regions advection is driving the negative temperature response in large parts of Europe in winter.

Considering the total number of CSs connected to blocking in CanESM2 (similar to the work of Pfahl and Wernli (2012) for summer but without the explicit colocation criterion), we find that up to 30%, 40%, and 50% of CSs coincide with blocked days in the Greenland, North Atlantic, and Scandinavian regions, respectively. Note, however, that the blocking regions are not exclusive, and a block may occur in more than one region simultaneously. Therefore, up to 70% of winter CSs in central Europe coincide with a blocking anywhere between 60°W and 30°E.

### 4.2 Blocking Impacts on Present Summer Heat Waves

**Figure 3:**
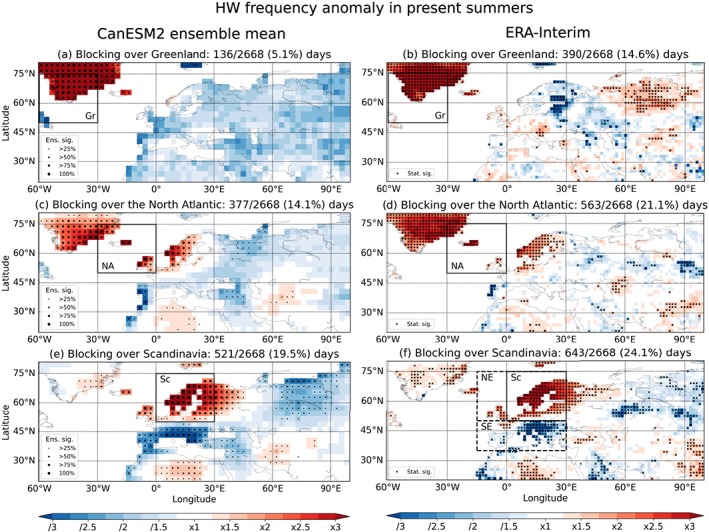
Same as Figure 2 but for summer (June–August) heat waves. The dashed boxes in (f) indicate northern Europe (NE) and southern Europe (SE) regions used in section 4.3.

Figure 3 shows the impact of blocking in the three regions on the HW frequency throughout Europe during present‐day summers (June–August). For blocking in the Greenland region no significant signal in the HW frequency is found in CanESM2 (Figure 3a). ERA‐Interim shows even weaker anomalies throughout most of Europe for blocking over Greenland. There is, however, also a distinct region of statistically significantly decreased HW frequencies in eastern Scandinavia (Figure 3b). Blocking in the North Atlantic region is correlated to HWs on the British Isles and Scandinavia, increasing the HW frequency by about a factor 2 for CanESM2 and ERA‐Interim (Figures 3c and 3d). On the Iberian Peninsula, an anticorrelation is found with blocking over the North Atlantic with HW frequencies reduced by up to one third. These findings for blocking over the North Atlantic are in good agreement with, for example, Sousa et al. (2018) who find positive temperature anomalies on the British Isles and southeastern Scandinavia as well as negative temperature anomalies on the Iberian Peninsula during blocking in this region.

A very distinct split in the HW response is visible between northern and southern Europe for blocking over Scandinavia. The HW occurrence in Scandinavia is increased by more than a factor of 3 and statistically significant across all ensemble members, during blocking over Scandinavia. In sharp contrast, southern Europe shows a decrease in HW frequency by a factor of 3, however, with fewer ensemble members showing statistical significance. The response pattern shows excellent agreement between CanESM2 (Figure 3e) and ERA‐Interim (Figure 3f), except for the Iberian peninsula, where about 50% of CanESM2 ensemble members show a statistically significant decrease in the HW frequency, while ERA‐Interim shows hardly any signal. This split can be attributed to different processes driving the temperature response. As has been shown by Sousa et al. (2018) for summer blocking over Scandinavia, diabatic processes play a dominant role at the location of the block and there are strong colocated positive radiation anomalies during blocking over this region. Since we define blocking as stationary high pressure at midlatitudes following a classical definition (see section 2.2), we only consider cases northward of 50°N. Blocking can hence not have the above mentioned direct effect for southern Europe (the effect of low‐latitude atmospheric ridges on southern Europe is addressed in Sousa et al. (2018)). Therefore, advection of cooler air from the north due to the anticyclonic motion of the block is most probably again the main driver of the strongly reduced HW frequency in southern Europe similar to the winter CS cases.

In total more than 60% of Scandinavian summer HWs are connected to blocking over Scandinavia in the CanESM2 ensemble mean. For ERA‐Interim the value reaches 80%, consistent with findings of Pfahl and Wernli (2012). At the same time, less than 10% of HWs in southern Europe co‐occur with blocking in the Scandinavian region, highlighting again the anticorrelation between blocking and HWs in some parts of Europe also in summer.

### 4.3 Present and Future European Extreme Temperature Response to Blocking Across Seasons

In the following we consider area averages over northern and southern Europe (as indicated in Figure 3f) and investigate ensemble mean and spread (see section 2.3 for details). This approach is motivated by the distinct split in the HW response to blocking over Scandinavia in present‐day summers (Figures 3e and 3f), which is also found in future summers (Figure S9) as well as in present and future spring (Figures S3 and S8), fall (Figures S6 and S10), and to some extent in winter (Figures S1 and S7). A split can also be found for the CS response to blocking over Scandinavia, particularly for ERA‐Interim and present‐day conditions in CanESM2 (Figures 2, S2, S4, and S5).

**Figure 4:**
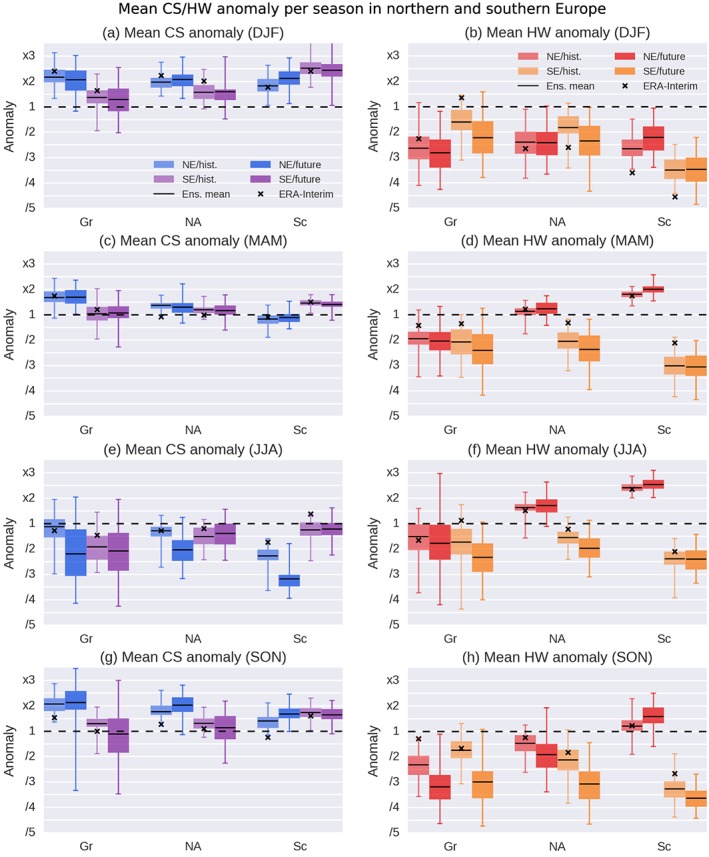
Cold spell (a, c, e, g) and heat wave (b, d, f, h) frequency anomaly in northern (blue, red) and southern (purple, orange) Europe during blocking in different regions (compare Figure 3) for winter (DJF), spring (MAM), summer (JJA), and fall (SON; from top to bottom) in the periods 1981–2010 (light shading) and 2070–2099 (dark shading). Shown is the 25th to 75th percentile range (colored boxes), the full range (whiskers), and the mean (black line) over all 50 ensemble members as well as ERA‐Interim (× sign). Gr = Greenland; NA = North Atlantic; Sc = Scandinavia; CS = cold spell; HW = heat wave; DJF = December–February; MAM = March–May; JJA = June–August; SON = September–November.

Figure 4 provides a summary on the dependence of the European CS and HW frequency on the region of atmospheric blocking during present‐day and future conditions. In the following we discuss all seasons, addressing (i) the CanESM2 performance compared to ERA‐Interim, and (ii) the temperature response to blocking in different regions for present and future conditions. Corresponding maps can be found in the supporting information (Figures S1–S6).

Regarding the impact of winter blocking on CSs, CanESM2 and ERA‐Interim are in good agreement (Figures 4a and 4b). Larger deviations are found for HWs, with a general underestimation in CanESM2. The difference in the sign of the HW anomaly between CanESM2 and ERA‐Interim for southern Europe during blocking over Greenland is based only on nonsignificant anomalies (Figure S1). The strong negative HW anomaly in northern and southern Europe during blocking over Scandinavia seen in ERA‐Interim is captured by none of the 50 CanESM2 ensemble members, while the spatial distribution compares well between reanalysis and model.

Hardly any changes are apparent in the winter CS and HW response to blocking in the future compared to the present, so that blocking can be expected to play the same important role in the development of winter cold extremes also in a warmer climate.

For spring CSs the ensemble interquartile range is notably narrow indicating excellent agreement on the blocking‐CS link across most ensemble members (Figures 4c and 4d). The response is also in good agreement with ERA‐Interim for present‐day conditions, except for North Atlantic blocking and northern Europe CSs, where ERA‐Interim shows a decrease in CSs for the northernmost part of Scandinavia (Figure S2).

Regarding HWs, good agreement in northern Europe is found, while in southern Europe HWs are underestimated by most CanESM2 ensemble members. Present and future spring HWs show a particularly strong sensitivity to the blocking region, similar to summer (Figure 3). Blocking over Greenland leads to a decreased HW frequency with a large ensemble spread. Blocking over the North Atlantic and Scandinavia has an opposing effect on northern and southern Europe: HWs in northern Europe increase during blocking by a factor of 2 in the area mean, while HWs in southern Europe decrease to one third. This strong sensitivity of the temperature response to the exact location of the block highlights the importance of further looking into atmospheric dynamics in spring as has been done, for example, by Cassou and Cattiaux (2016).

In present summers, both CS and HW anomalies in CanESM2 are in good agreement with ERA‐Interim (Figures 4e and 4f). However, during blocking over Greenland they show a large spread across ensemble members, with the ensemble means indicating a tendency toward lower temperatures. As discussed in section 4.2, blocking over the North Atlantic and Scandinavia has the opposite effect on northern and southern Europe. This effect is strongest during blocking over Scandinavia, increasing the HW frequency in northern Europe almost by a factor of 2.5, while at the same time decreasing it in southern Europe by a factor of 2.5 during present day as well as future conditions (Figures 3 and S9).

For fall blocking (Figures 4g and 4h), the CS response is comparable to winter (Figure 4a), while the HW response is comparable to summer (Figure 4f). In particular, the split HW response for northern and southern Europe is also apparent in fall. Furthermore, fall is the only season where some of the future HW responses are different from present conditions in a significant number of ensemble members. It is, however, noteworthy that hardly any of the (present or future) responses to blocking over Greenland and the North Atlantic are statistically significant in the spatially resolved view (Figures S6 and S10).

## 5 Discussion and Conclusions

We used a large, 50‐member ensemble of the CanESM2 model to investigate the impact of blocking high‐pressure systems in different regions and seasons on temperature extremes in Europe during present and future conditions.

For present‐day conditions (1981–2010), the link between blocking and extreme temperatures is well represented despite an underestimation of blocking occurrence in CanESM2 compared to ERA‐Interim for most seasons and regions. For the future (2070–2099), the ensemble mean shows a robust decrease in the number of blocked days over Greenland and the North Atlantic, while no significant trend is apparent over Scandinavia. These findings are in agreement with recent research on the evolution of blocking under climate change (e.g., Kennedy et al., 2016; Matsueda & Endo, 2017).

We find blocking to be linked to warm conditions in summer and—to a certain degree—also in spring and fall, with blocking significantly increasing the HW frequency in northern Europe during those seasons. This is in agreement with earlier studies connecting blocking to an increase in colocated summer HWs, which is found to be mainly driven by increased radiative forcing at the location of the block (e.g., Meehl & Tebaldi, 2004; Pfahl & Wernli, 2012; Sousa et al., 2018). However, our results also show anticorrelation of blocking with HWs in southern Europe for most seasons and regions. This dual impact of blocking on European HWs is consistent with a combination of increased radiative heating in the region of the block and favored cold advection on the eastern and southern flanks as discussed in section 4.2. In this regard our findings also show that the response of northern European HWs to blocking highly depends on the region of the block in spring, summer, and fall. Blocking west of Europe over Greenland tends to lower the HW frequency in northern Europe, while blocking further east over Scandinavia increases it.

The blocking impact on CSs is found to be strongest in winter, consistent with the literature (e.g., Buehler et al., 2011; Sillmann et al., 2011). Blocking anywhere in the Euro‐Atlantic region increases the CS frequency in all of Europe in winter and mostly also in spring and fall, mainly driven by cold advection (see section 4.1). In summer, blocking conversely leads to a decrease in the occurrence of CSs as radiative effects become more important as also noted by Sousa et al. (2018).

Despite the general underestimation of blocking in CanESM2, the link between present‐day blocking and HW and CS occurrences in Europe is found to be well represented compared to ERA‐Interim in most of the investigated regions, seasons, and ensemble members. This is in agreement with findings by Masato et al. (2014) and gives confidence in model‐based impact studies, which, for example, assess associated risks (Zscheischler & Seneviratne, 2017). For the future we find that the blocking link to extreme temperatures is essentially preserved, indicating that blocking will continue to play an essential role in the development of CSs and HWs (e.g., Schaller et al., 2018). Our work therefore shows that continued research to increase the understanding of the physical mechanisms behind the link between blocking and both HWs and CSs, not only in summer and winter but in all seasons, is essential for predicting future climate impacts and associated risks.

## Supporting information



Supporting Information S1
