# Supplementary material for: Dependence of Present and Future European Temperature Extremes on the Location of Atmospheric Blocking
Source: Geophys Res Lett. 2018 Jun 28;45(12):6311–20. doi: 10.1029/2018GL077837 (PMC6190735; doi:10.1029/2018GL077837)
Supplement: Supplementary file 1 — Supporting Information S1 [file GRL-45-6311-s001.pdf]

Supporting Information for "Dependence of present and future European heat waves and cold spells on the location of atmospheric blocking"

Lukas Brunner[*1,2], Nathalie Schaller[3], James Anstey[4], Jana Sillmann[3], Andrea K. Steiner[1,2,5]

[1]Wegener Center for Climate and Global Change (WEGC), University of Graz, Graz, Austria
[2]FWF-DK Climate Change, University of Graz, Graz, Austria
[3]Center for International Climate Research (CICERO), Oslo, Norway
[4]Canadian Centre for Climate Modelling and Analysis, Environment and Climate Change Canada, University of Victoria, Victoria, Canada
[5]Institute for Geophysics, Astrophysics, and Meteorology, Institute of Physics, University of Graz, Graz, Austria
[*]Now at Institute for Atmospheric and Climate Science, ETH Zurich, Zurich, Switzerland

Contents of this file

1. Table S1
2. Figures S1 to S10

Introduction

Table S1 provides the underlying blocking statistics used in Fig. 1.

Figures S1 to S 10 provide additional maps providing more details for the results presented in Fig. 4.

Corresponding author: Lukas Brunner, lukas.brunner@env.ethz.ch

Table S1. Percentage of blocked days per season and region for ERA-Interim and the CanESM2 ensemble mean and ensemble standard deviation during present and future conditions. *Italics* for CanESM2 present: ERA-Interim frequency lies within one standard deviation of the CanESM2 ensemble mean. **Bold** for CanESM2 future: standard deviations of present and future periods do not overlap.

Season	Loc.	ERAI present	CanESM2 present	CanESM2 future
DJF	Gr	13.6 %	10.3 % ± 1.6 %	**6.3 % ± 1.2 %**
	NA	17.0 %	13.6 % ± 1.9 %	**10.0 % ± 1.5 %**
	Sc	18.7 %	13.2 % ± 1.7 %	11.9 % ± 1.7 %
MAM	Gr	19.3 %	15.3 % ± 1.9 %	**11.7 % ± 1.6 %**
	NA	23.5 %	23.8 % ± 1.9 %	**18.9 % ± 2.3 %**
	Sc	25.0 %	26.4 % ± 2.2 %	24.3 % ± 2.4 %
JJA	Gr	14.6 %	5.1 % ± 1.2 %	3.1 % ± 0.9 %
	NA	21.1 %	14.1 % ± 1.7 %	**10.7 % ± 1.4 %**
	Sc	24.1 %	19.5 % ± 1.9 %	18.1 % ± 2.0 %
SON	Gr	16.9 %	10.8 % ± 1.4 %	**4.7 % ± 1.2 %**
	NA	21.5 %	14.1 % ± 1.6 %	**8.1 % ± 1.3 %**
	Sc	20.3 %	16.0 % ± 1.6 %	15.2 % ± 2.1 %

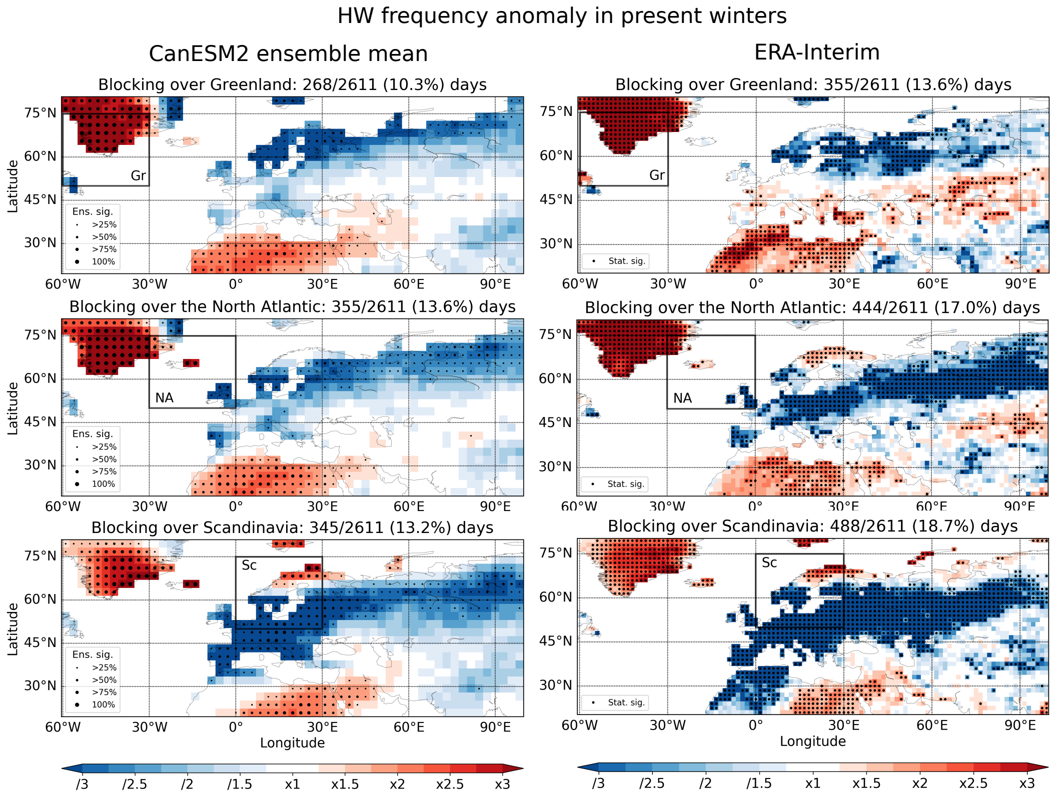

Figure S1. Heat wave frequency anomaly during blocking in different regions (gray box) for winters (DJF) in the period 1981 to 2010 for the CanESM2 ensemble mean (left) and ERA-Interim (right). Statistical significance at the 10 % (2-sided) level is indicated by dots, the dot size gives the number of ensemble members which show significance for CanESM2.

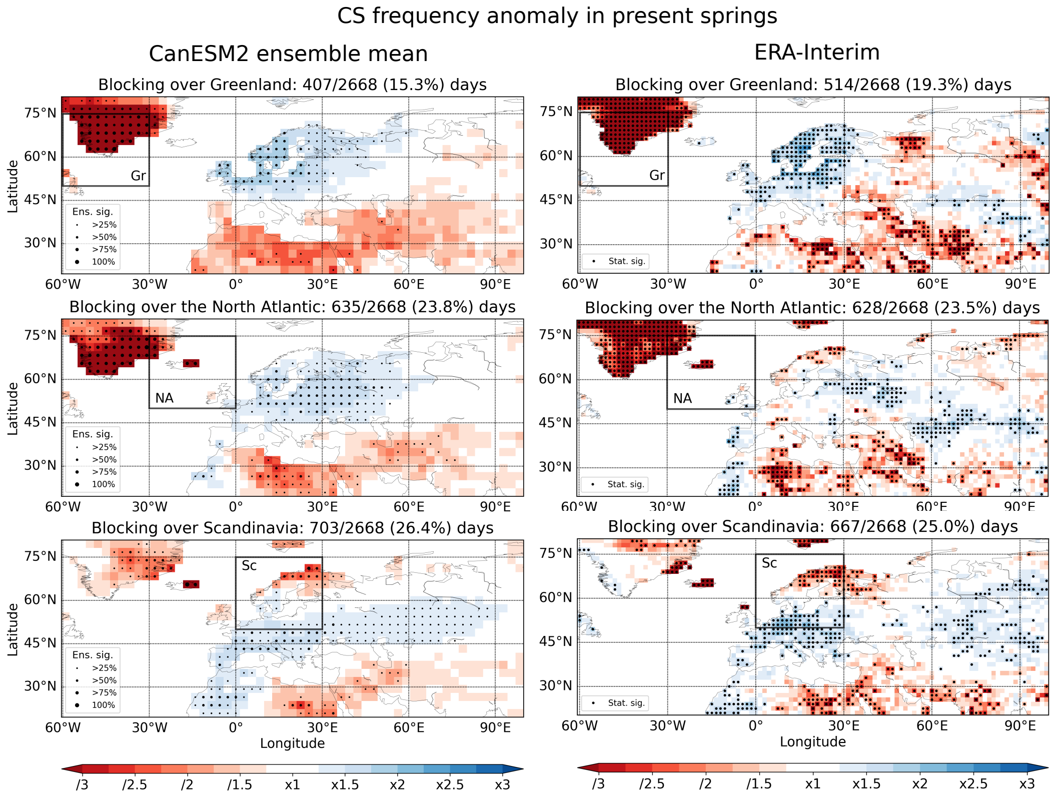

Figure S2. Cold spell frequency anomaly during blocking in different regions (gray box) for springs (MAM) in the period 1981 to 2010 for the CanESM2 ensemble mean (left) and ERA-Interim (right). Statistical significance at the 10 % (2-sided) level is indicated by dots, the dot size gives the number of ensemble members which show significance for CanESM2.

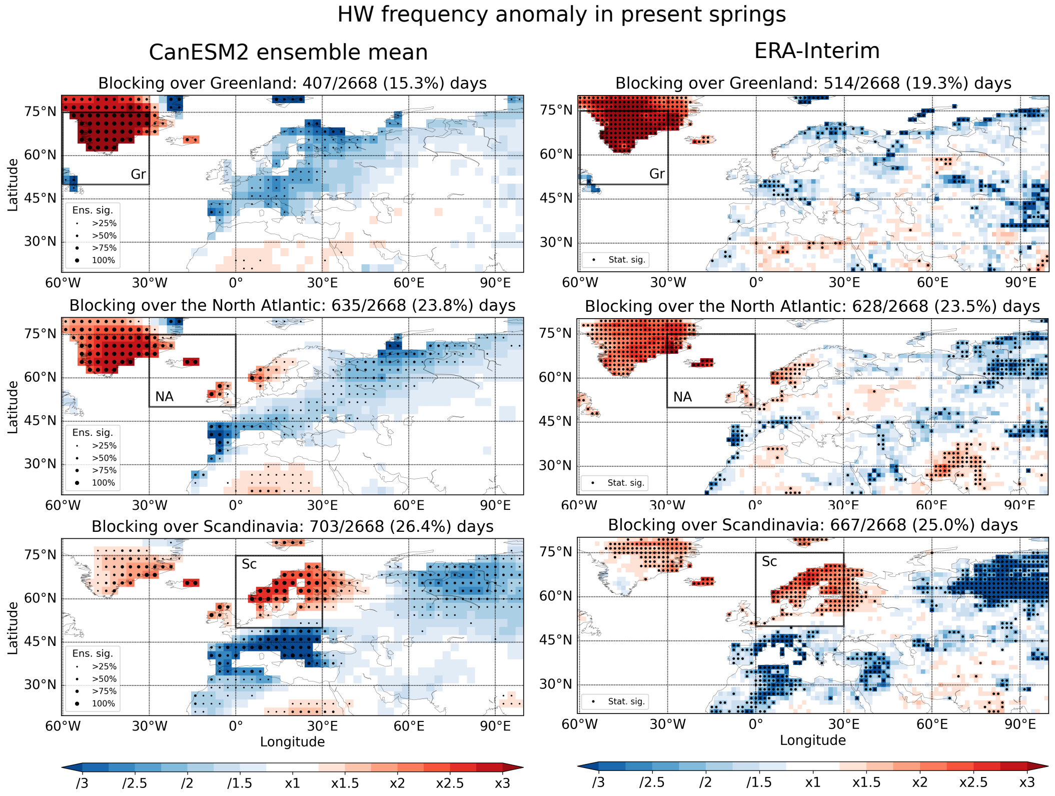

Figure S3. Heat wave frequency anomaly during blocking in different regions (gray box) for springs (MAM) in the period 1981 to 2010 for the CanESM2 ensemble mean (left) and ERA-Interim (right). Statistical significance at the 10 % (2-sided) level is indicated by dots, the dot size gives the number of ensemble members which show significance for CanESM2.

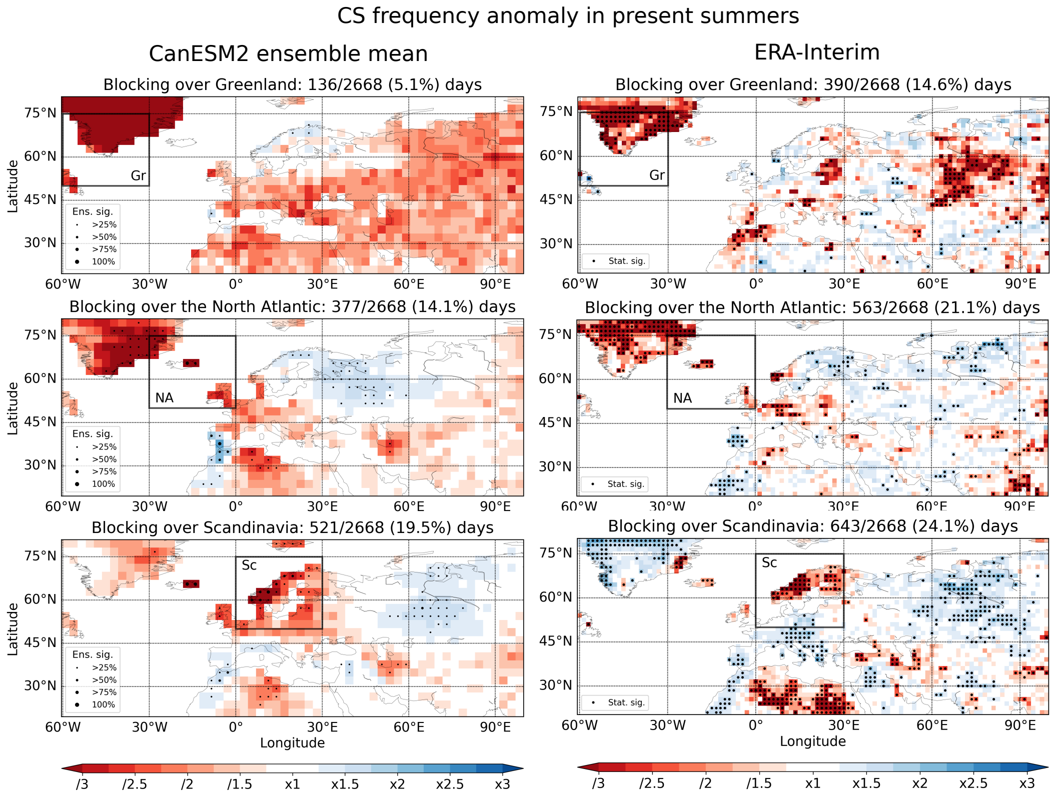

Figure S4. Cold spell frequency anomaly during blocking in different regions (gray box) for summers (JJA) in the period 1981 to 2010 for the CanESM2 ensemble mean (left) and ERA-Interim (right). Statistical significance at the 10 % (2-sided) level is indicated by dots, the dot size gives the number of ensemble members which show significance for CanESM2.

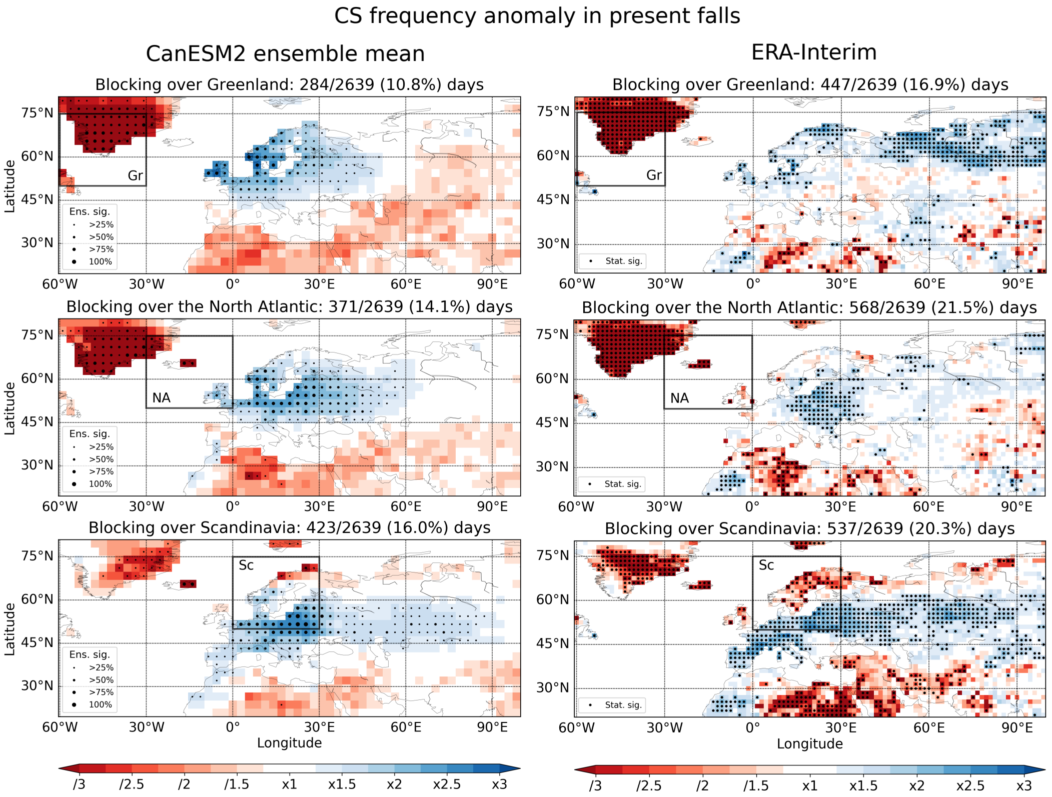

Figure S5. Cold spell frequency anomaly during blocking in different regions (gray box) for falls (SON) in the period 1981 to 2010 for the CanESM2 ensemble mean (left) and ERA-Interim (right). Statistical significance at the 10 % (2-sided) level is indicated by dots, the dot size gives the number of ensemble members which show significance for CanESM2.

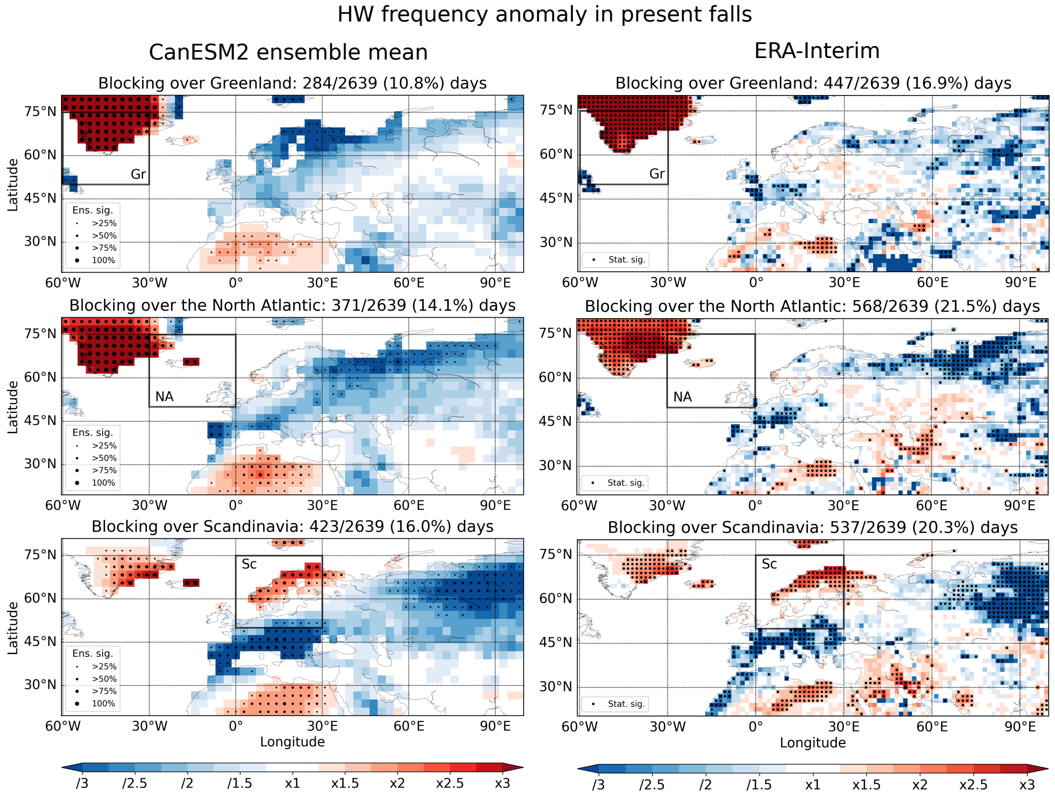

Figure S6. Heat wave frequency anomaly during blocking in different regions (gray box) for falls (SON) in the period 1981 to 2010 for the CanESM2 ensemble mean (left) and ERA-Interim (right). Statistical significance at the 10 % (2-sided) level is indicated by dots, the dot size gives the number of ensemble members which show significance for CanESM2.

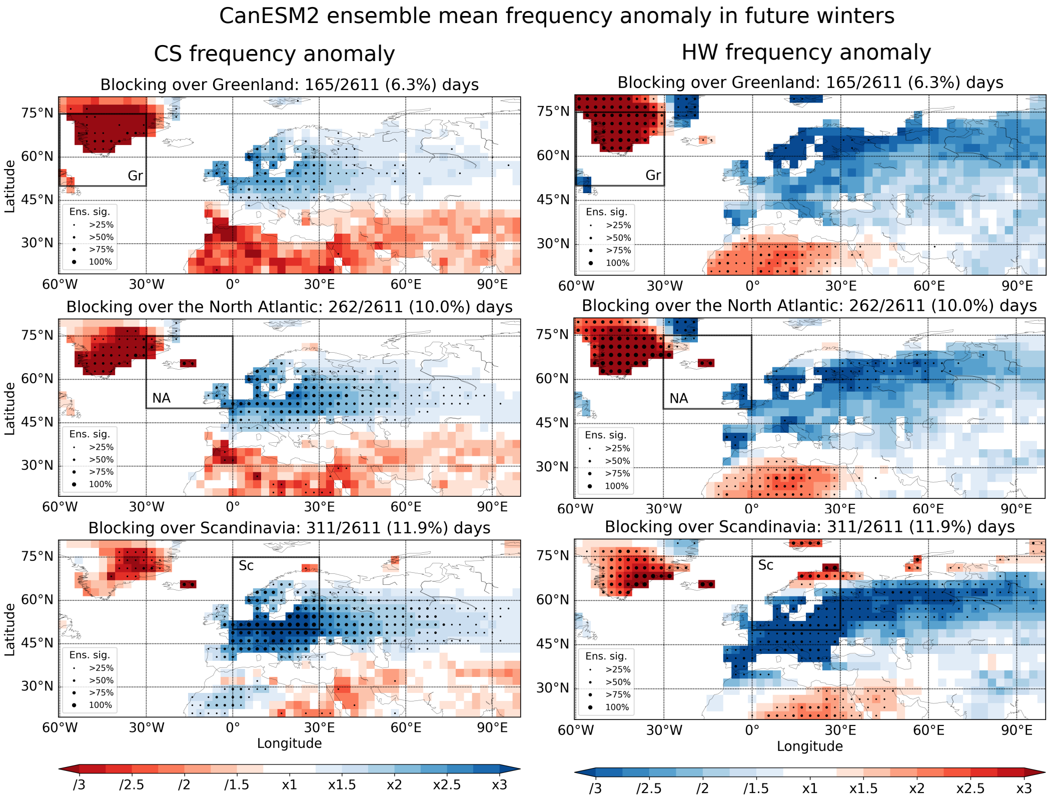

Figure S7. Cold spell (left) and heat wave (right) frequency anomaly during blocking in different regions (gray box) for winters (DJF) in the period 2070 to 2099 for the CanESM2 ensemble mean. Statistical significance at the 10 % (2-sided) level is indicated by dots, the dot size gives the number of ensemble members which show significance.

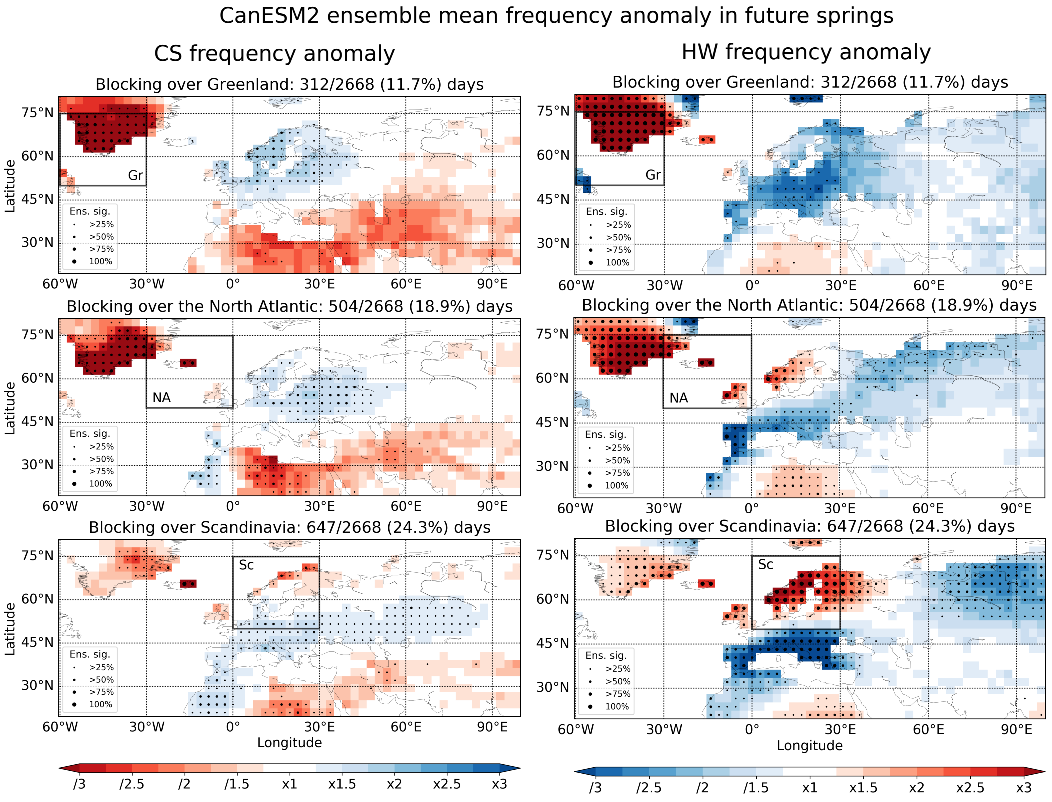

Figure S8. Cold spell (left) and heat wave (right) frequency anomaly during blocking in different regions (gray box) for springs (MAM) in the period 2070 to 2099 for the CanESM2 ensemble mean. Statistical significance at the 10 % (2-sided) level is indicated by dots, the dot size gives the number of ensemble members which show significance.

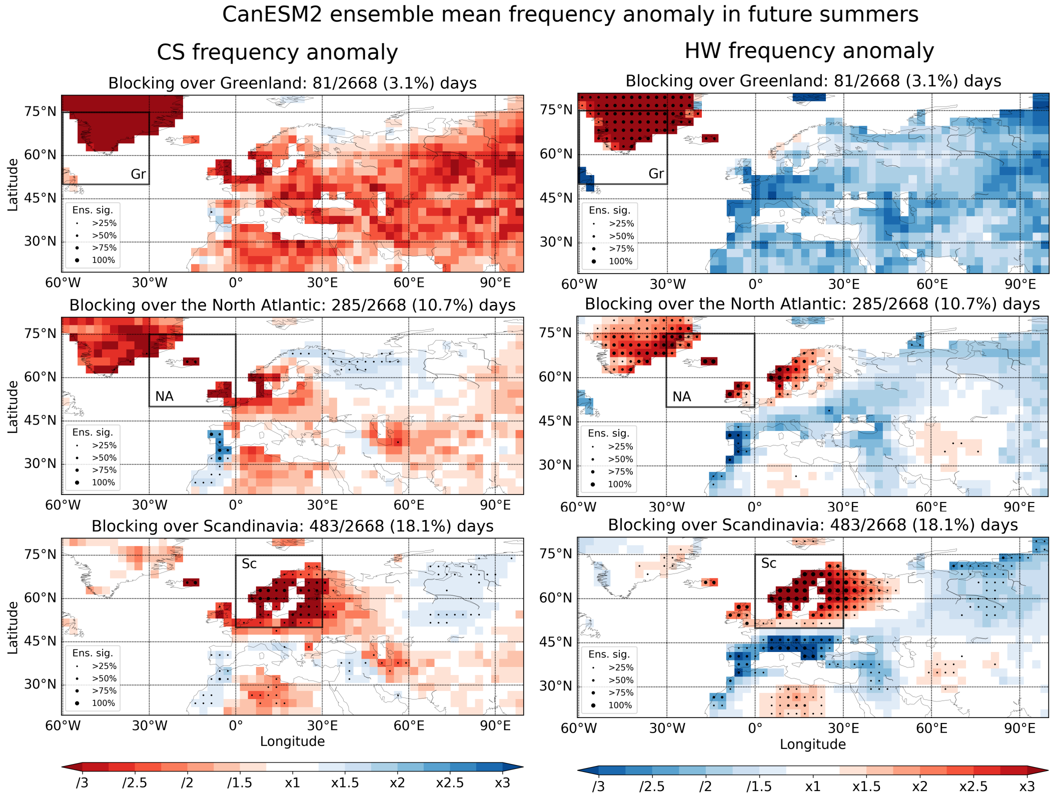

Figure S9. Cold spell (left) and heat wave (right) frequency anomaly during blocking in different regions (gray box) for summers (JJA) in the period 2070 to 2099 for the CanESM2 ensemble mean. Statistical significance at the 10 % (2-sided) level is indicated by dots, the dot size gives the number of ensemble members which show significance.

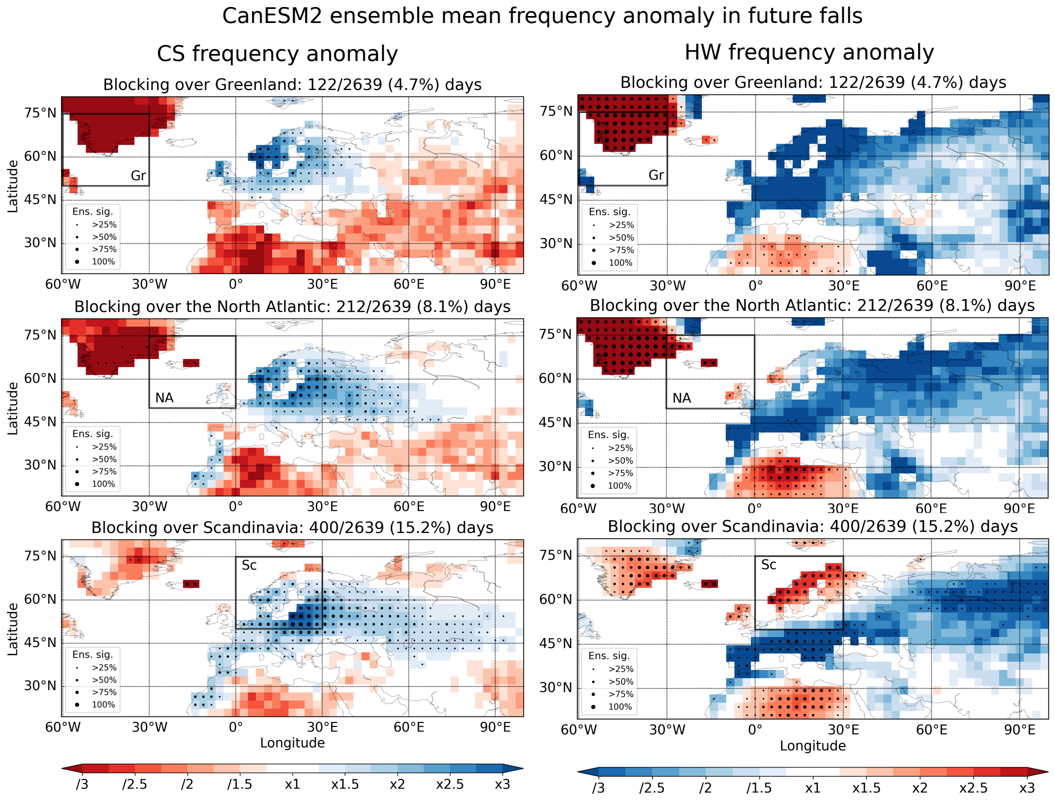

Figure S10. Cold spell (left) and heat wave (right) frequency anomaly during blocking in different regions (gray box) for falls (SON) in the period 2070 to 2099 for the CanESM2 ensemble mean. Statistical significance at the 10 % (2-sided) level is indicated by dots, the dot size gives the number of ensemble members which show significance.
